# *Drosophila* photoreceptor tethering by a laminin-Eys scaffold

**DOI:** 10.1016/j.isci.2025.112732

**Published:** 2025-05-22

**Authors:** Donald F. Ready, Henry C. Chang

**Affiliations:** 1 Department of Biological Sciences, Purdue University, 915 Mitch Daniels Boulevard, West Lafayette, IN 47907-2054, USA

**Keywords:** Mechanobiology, Cell biology, Biophysics

## Abstract

Visual acuity in *Drosophila* requires precise photoreceptor alignment along the optical axis, maintained by longitudinal tension between a rigid cornea and a contractile retinal base. Here, we identify the rhabdomere caps—an extracellular matrix (ECM) structure that links rhabdomere tips to the integrin-decorated basal surfaces of overlying, lens-forming cone cells. Rhabdomere caps form perlecan-filled peaks shaped by a trapezoidal LanB1 (laminin) grid, which mirrors the inter-rhabdomeral space (IRS) contour. Our study revealed that Eys (*eyes shut*), a photoreceptor-secreted proteoglycan essential for IRS formation, guides LanB1 and perlecan deposition by cone cells during pupal development. Disruption of LanB1 results in rhabdomere tip detachment, IRS collapse, and impaired tension transmission. These findings reveal that cone cells and photoreceptors collaboratively sculpt a rigid LanB1 grid that caps and reinforces the distal IRS lumen. This composite ECM structure preserves rhabdomere organization and evenly distributes mechanical forces, ensuring photoreceptor alignment and optical fidelity.

## Highlights

• Rhabdomere caps are ECM specializations that tether photoreceptors to cone cells
• Rhabdomere caps contain perlecan-rich peaks outlined by a laminin β1 (LanB1) grid
• Photoreceptors secrete Eys to pattern the LanB1 grid at the distal IRS
• The LanB1 grid shields photoreceptor ends from deformation during morphogenesis

## Graphical abstract

**Figure:**
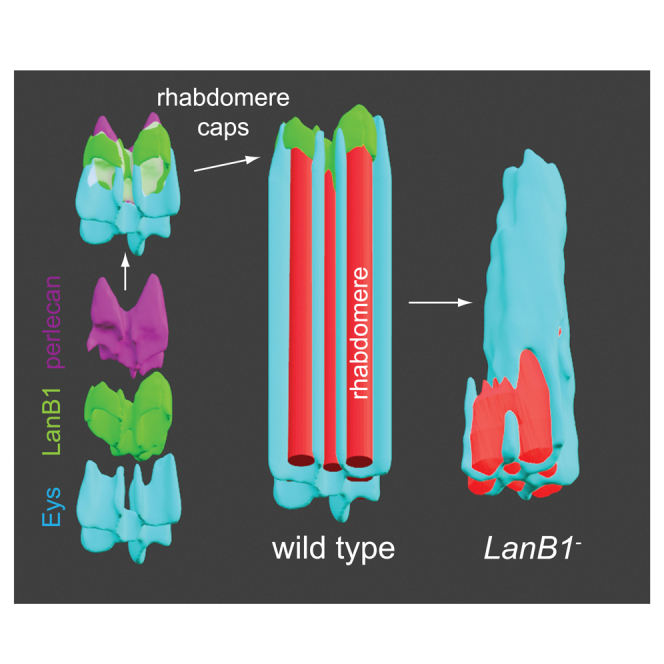

## Introduction

Accurate visual perception depends on the precise positioning of photodetectors along the retina’s optical axis. In vertebrate camera-like eyes, photosensory outer segments are aligned to this axis by tension perpendicular to the layered retina and retinal pigment epithelium at the eye’s curved posterior.^1^ In dome-like compound eyes, photosensory rhabdomeres are stretched between a rigid, faceted cornea, and a convex basement membrane.^2^ In both eye types, the cellular and molecular structures anchoring photodetectors must be precisely constructed to effectively transmit tension for alignment. Extracellular matrix (ECM) structures, with their diverse compositions, contours, and mechanical properties, play crucial roles as intermediates in force transmission during development.^3^^,^^4^^,^^5^^,^^6^^,^^7^ However, how retinal ECM is assembled and sculpted to achieve photosensor arrangement remains largely unknown.

**Figure 1:**
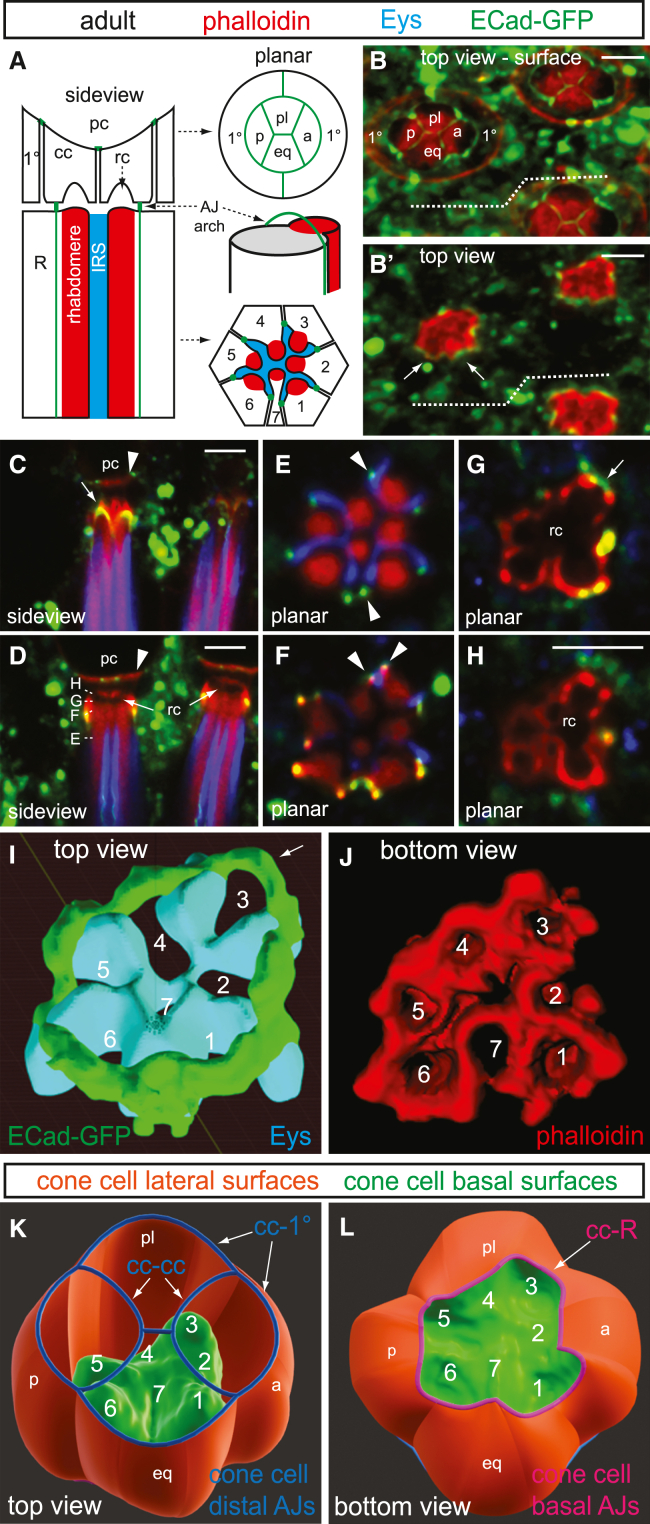
Adherens junctions and rhabdomere caps connect photoreceptors to cone cells (A–H) Sideview and cross-section schematics depict the photoreceptor and cone cell organization in one ommatidium. At the distal end, the cone cells (a, anterior; eq, equatorial; p, posterior; pl, polar) apically form H-shaped adherens junctions (green) in the center and contact the pseudocone (pc) and are laterally surrounded by two primary pigment cells (1°). Underneath, the photoreceptors (R cells, only R1-7 are shown in the cross-section) contain cylindrical rhabdomeres (red) separated by intra-rhabdomeral space (IRS, blue) and form longitudinal adherens junctions, seen as dots at the IRS tips in the cross-section. The distal tips of rhabdomeres are anchored to the cone cell basal surfaces via rhabdomere caps (rc), which are ECM peaks enclosed by arch-like adherens junctions at the photoreceptor-cone cell boundary. Opacity (B) and intensity (B′) rendered images, along with confocal sections (C–H) of adult *ECad-GFP* retinas stained with phalloidin (red) and αEys antibody (blue; omitted in 3D renderings for clarity). The equator is indicated by a dashed line. Grazing (C) and mid-ommatidial (D) side views, along with cross-sections (E–H; depths indicated in D), detail the junctional architecture at the distal photoreceptor-cone cell border. Anterior is to the right. Scale bar, 5 μm. (I) A 3D model of E-Cad (green) fence atop the IRS (Eys, blue). (J) An ImageJ 3D Viewer surface rendering of F-actin (red) lining the rhabdomere caps viewed from below. (K and L) A 3D model showing the cone cell basal surfaces (green) contacting rhabdomere caps protruding from below (K: top view; L: bottom view). In the top view, blue outlines the cone cell apical adherens junctions, as seen with ECad (B). In the bottom view, magenta outlines the ECad arches marking the cone cell-photoreceptor boundary. cc-cc, cc-1°, and cc-R indicate cone cell-cone cell, cone cell-primary pigment cell, and cone cell-photoreceptor junctions, respectively. Lateral cone cell surfaces are shown in orange. Not shown are the thin inter-retinular fibers that extend proximally from each cone cell body to the retinal floor where they intercalate between the photoreceptors to form the cone cell plate.

The *Drosophila* eye provides an excellent model for studying how ECM dynamics and contractile forces drive tissue morphogenesis. The compound eye consists of approximately 700 unit eyes (ommatidia), each containing eight centrally located photoreceptors arranged around an ECM-filled central lumen called the inter-rhabdomeral space (IRS, Figure 1A). Retinal cell pattern formation begins with the sequential recruitment of photoreceptors behind the morphogenetic furrow, an apical indentation that sweeps across the larval eye disc.^8^ The IRS emerges early in pupal development as photoreceptor apical surfaces make an inward turn, forming a closed lumen.^9^ This lumen is expanded during mid-pupal development by Eys (Eyes shut), an agrin/perlecan-related protein secreted by photoreceptors,^10^^,^^11^ forming an ommatidial core that extends throughout the retinal depth (∼100 μm by eclosion). The IRS optically insulates the rhabdomeres, enabling their function as waveguides and supporting the open rhabdom/neural superposition design of the fly eye.^2^ As the IRS extends along the optical axis, it ensures precise positioning of rhabdomere distal tips beneath the lens-secreting cone cells, facilitating accurate light focusing.

The photoreceptor clusters are surrounded by a honeycomb lattice of interommatidial cells (IOCs).^12^ At the retinal floor, actin stress fibers within the IOC endfeet contract to reduce floor area, generating the eye’s panoramic convex curvature.^9^^,^^13^ This tissue shape change is mediated by a tensile network composed of collagen-fortified grommets symmetrically connected to IOC stress fibers, assembled through sequential and cell-specific ECM depositions.^14^ ILK (integrin-linked kinase), a component of the IPP complex (ILK, Parvin, and PINCH) that links actomyosin cytoskeletal elements to integrin,^15^^,^^16^ tethers IOC stress fibers to the grommets along three axes.^14^ These stress fiber contractions are coordinated by intercellular Ca^2+^ waves propagating across the IOC network.^17^

The contraction of IOC stress fibers at the retinal floor generates longitudinal tension that stretches each ommatidium’s rhabdomeres taut along the optical axis.^13^^,^^18^ The transmission of this longitudinal force relies on tethering of rhabdomere tips. The four lens-secreting cone cells, also known as Semper cells, are well positioned to tether these tips, as they apically cover the photoreceptor surfaces and extend their basal endfeet to form the cone cell plate (CCP), which serves as the proximal anchor for rhabdomeres.^9^ Cone cell-expressed ILK localizes to the junctions with rhabdomere tips, and rhabdomeres selectively detach from the basal side in tissues lacking integrin or ILK.^9^^,^^14^ This selective detachment suggests that additional mechanism(s) independent of integrin must tether rhabdomere tips at the distal side. While integrins and their associated complexes are implicated, the specific ECM ligand and structure responsible for rhabdomere alignment remain unknown. Furthermore, the cone cells’ quartet arrangement differs from the trapezoidal organization of rhabdomeres, raising questions about how forces from cone cells are distributed across these discrete photoreceptors.

In this study, we characterize the composition, assembly, and function of rhabdomere caps—an ECM intermediate positioned between rhabdomere distal tips and cone cell basal surfaces. Rhabdomere caps consist of perlecan-filled peaks delineated by a LanB1 grid. Mutational analyses reveal that this LanB1 grid is crucial for rhabdomere alignment. Disruption of the LanB1 grid leads to tears at rhabdomere distal tips and deformation of the distal IRS lumen, underscoring the importance of ommatidial core integrity for tension transmission. Genetic evidence suggests that photoreceptors and cone cells collaborate to pattern LanB1 deposition in the shape of the IRS, forming a grid that caps and reinforces the distal IRS lumen. This rigid ECM grid immobilizes rhabdomere distal tips, ensuring uniform tensile force distribution along the optical axis. Thus, beyond its optical role, the IRS acts as a tension-shielding scaffold that absorbs and redistributes mechanical forces imposed by IOC-driven stress fiber contraction. By buffering against these forces, the ommatidial core preserves rhabdomere structural integrity and ensures precise optical axis alignment.

## Results

### Adherens junctions and rhabdomere caps anchor photoreceptor distal ends

To understand how rhabdomeres are anchored at their distal ends, we used *ECad-GFP* (*E-Cadherin*; *shotgun*)^19^ and *Fas3-GFP* (*Fasciclin 3*)^20^ to label adherens junction (AJs) and septate junction (SJ) at the photoreceptor/cone cell boundary. An opacity-rendered image of an adult *ECad-GFP* retina reveals an array of concave “bowls” formed by the apical surfaces of cone cells and surrounding primary pigment cells (1°, Figure 1B). These bowls are complementary with the overlying lens element, the pseudocone (pc, Figures 1A, 1C, and 1D)—a vitreous gel secreted by cone cells that, along with the cornea, focuses light onto the distal tips of the photosensitive rhabdomeres.^21^ At the interface between cone cells and the pseudocone, F-actin forms a planar plate-like contact (arrowheads, Figures 1C and 1D). Centrally, the apical membranes of the cone cells converge in an H-shaped AJ where the polar and equatorial cone cells meet at the center (Figures 1A and 1B, Video S1). Peripherally, cone cell AJs connect with primary pigment cells (Figures 1A and 1B), aligning them with the global eye axes. Fas3-GFP-labeled SJs form a similar H-shape beneath the AJs (Figure S1).^22^ An intensity-rendered view below the apical surface highlights a strong F-actin trapezoid encircling the seven rhabdomere tips (Figure 1B’).

Video S1. Junctional organization at the distal region of adult retina, related to Figure 1 3D rendering (Volocity, PerkinElmer) of an *ECad-GFP* cluster stained with phalloidin (red) and αEys antibody (blue).

Longitudinal and tangential sections of the adult photoreceptor/cone cell junction reveal a sculpted extracellular lumen, surfaced distally by cone cell basal membranes and proximally by photoreceptor apical membranes. This lumen is shaped by AJs, which transition from photoreceptor-photoreceptor junctions to photoreceptor-cone cell junctions. In proximal cross-sections, the longitudinally oriented photoreceptor-photoreceptor AJs mark the boundary of IRS radial arms, appearing as dots flanking the rhabdomeres (arrowheads, Figure 1E). At the photoreceptor-cone cell boundary, each AJ between adjacent photoreceptors splits into two distinct AJs that link photoreceptors to the overlying cone cells (arrowheads, Figure 1F). In progressively distal cross-sections, ECad-GFP dots from either side of each rhabdomere draw closer together (arrow, Figure 1G), forming arches that close distally, completing the AJ defining the photoreceptor’s apical border. In 3D renderings (arrows, Figures 1B’ and 1I; Video S1) and side views (arrow, Figure 1C), these photoreceptor-cone cell AJs form a trapezoidal “fence” of ECad-GFP arches surrounding the rhabdomere tips. Each of the four cone cells individually contributes an AJ arch over specific photoreceptors: R2 (anterior), R4 (polar), R5 (posterior), and R7 (equatorial), creating the trapezoid’s flat edges (Figure 3P). In contrast, the arches over R1, R3, and R6 are composite structures formed by adjacent cone cells, marking the trapezoid’s corners. Phalloidin-stained stress fibers extend distally from these arches, converging above the arch peaks (Figure 1C), suggesting that stress fibers generate tension to shape the curvature of these arches.

Staining with αEys antibody reveals this extracellular lumen is a composite. Eys fills IRS, the proximal portion of this lumen (below the AJ arches), and contributes to the formation of an open rhabdom.^10^^,^^11^ At the plane of the AJ arches, the IRS transitions into rhabdomere caps—dome-like extracellular structures located above the distal tips of each rhabdomere (arrows, Figure 1D). Eys minimally intrudes into the rhabdomere caps, which remain continuous with the IRS but are distinct in both form and content. In tangential sections across the AJ arches, rhabdomere caps present as a trapezoidal cavity approximately 4 μm in diameter, enclosed by phalloidin-positive cone cell surfaces. Where the optical plane intersects an arch, ECad-GFP completes the perimeter (arrow, Figure 1G). In more distal cross-sections, rhabdomere caps appear as circular phalloidin profiles, shaped by cone cell basal membrane drawn over the caps (Figure 1H). These patterns depict the rhabdomere caps as interconnected extracellular peaks (∼1 μm in height) situated over rhabdomere tips, bounded peripherally by ECad arches and extending distally into the cone cells (Figures 1I–1L). The location and structure of these rhabdomere caps suggest they anchor the distal rhabdomere tips to the F-actin-rich basal surface of the cone cells, providing additional adhesion beyond that of the photoreceptor-cone cell AJs.

### Rhabdomere caps contain differentially localized perlecan and LanB1

To investigate the composition of rhabdomere caps, we examined the localization of various GFP-tagged basement membrane components in the adult retina, including *vkg::GFP* (collagen IV; viking), *trol-GFP*^*ZCL1700*^ (perlecan; terribly reduced optic lobes), and *LanB1-GFP* (laminin B1). *vkg::GFP* and *trol-GFP* are protein trap lines, each with an in-frame GFP splicing cassette inserted into the endogenous locus,^23^ while *LanB1-GFP* is a transgenic line containing an exogenous genomic fragment with GFP fused in-frame to the C-terminus of LanB1.^24^ *LanB1-GFP* rescues the lethality associated with *LanB1* loss-of-function mutations (*LanB1*^*SK1*^ and *LanB1*^*SK7*^), demonstrating its functionality.

**Figure 2:**
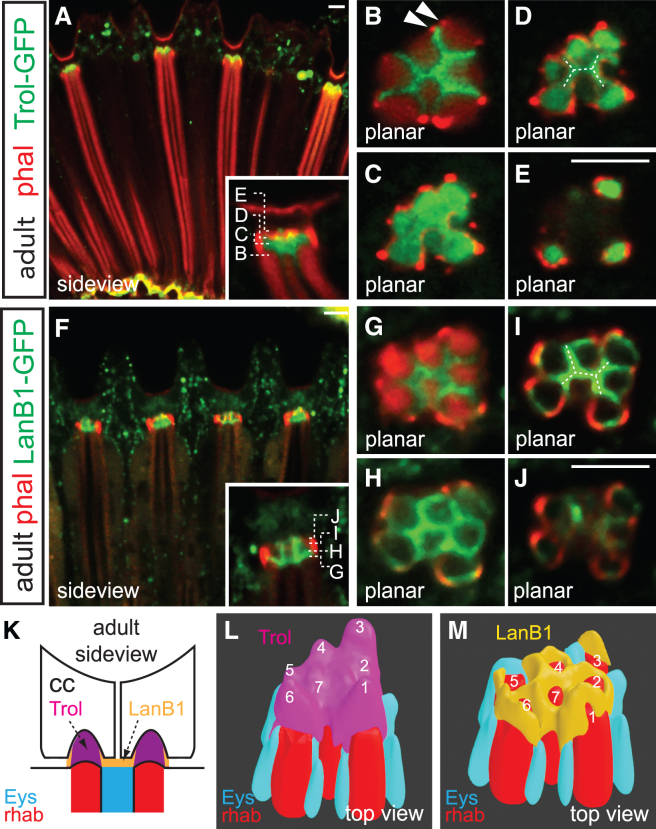
Rhabdomere caps contain differentially localized perlecan and laminin (A–M) Confocal micrographs of adult *trol-GFP* (A–E) and *LanB1-GFP* (F–J) retinas stained with phalloidin (red). Side views (A and F; higher magnifications shown in insets) and cross-sections (B–E, G–J; depths indicated in corresponding insets) detail the localization of these proteins in the rhabdomere caps. Anterior is to the right. Scale bar, 5 μm. A sideview schematic (K) and Blender model top views (L and M) display rhabdomeres (red), Eys (blue), and ECM components (Trol in purple and LanB1 in orange). Numbers indicate photoreceptor identity.

While collagen IV is prominent at the retinal basement membrane,^14^ Vkg::GFP is not present at the rhabdomere distal tips in both adult and P12 pupal retinas (Figure S2), indicating that collagen is not a component of the rhabdomere caps. In contrast, Trol-GFP is localized near the top of the IRS (Figures 2A and 2B; Video S2), where Eys is lost and photoreceptor AJs transition into AJ arches (arrowheads). In progressively distal cross-sections, Trol-GFP surrounds the distal rhabdomere tips, with limited overlap with Eys (not shown) (Figure 2B) before filling the shared space of the cap (Figure 2C), that then separates into individual rhabdomere caps (Figure 2D), with the tallest caps located above photoreceptors 1, 3, and 6 (Figure 2E). Trol-GFP forms an H-shaped figure at the central junction of cone cell basal membranes (dashed lines, Figure 2D) indicating that perlecan extends into a basal crevice at the cone cell boundary. Trol thus fills the entire extracellular volume between photoreceptors and overlying cone cells (Video S2), its distal surface reflecting the combined contour of the cone cell basal membranes (Figure 2L).

Video S2. Perlecan localization at adult rhabdomere caps, related to Figure 2 3D rendering (Volocity) of an adult *trol-GFP* cluster stained with phalloidin (red).

While Trol-GFP fills the rhabdomere caps, LanB1 displays a more patterned distribution within them (Figures 2F–2J; Video S3). Like Trol-GFP, LanB1-GFP localizes to the top of the IRS (Figure 2G). In distal cross-sections, LanB1-GFP delineates the lateral boundaries of the Trol-GFP-filled caps (Figures 2H–2J), forming a trapezoidal grid above the rhabdomere tips (Figure 2M). An H-shaped figure with increased LanB1-GFP at the grid center aligns basally with the IRS contour and distally with the cone cell boundary (dashed lines, Figure 2I). These distinctive patterns of LanB1 and perlecan (summarized in Figure 2K) suggest that the ECM materials in the RCs are discretely deposited and likely fulfill distinct roles.

Video S3. LanB1 localization at adult rhabdomere caps, related to Figure 2 3D rendering (Volocity) of an adult *LanB1-GFP* cluster stained with phalloidin (red).

### Integrin mediates cone cell-rhabdomere cap contact

**Figure 3:**
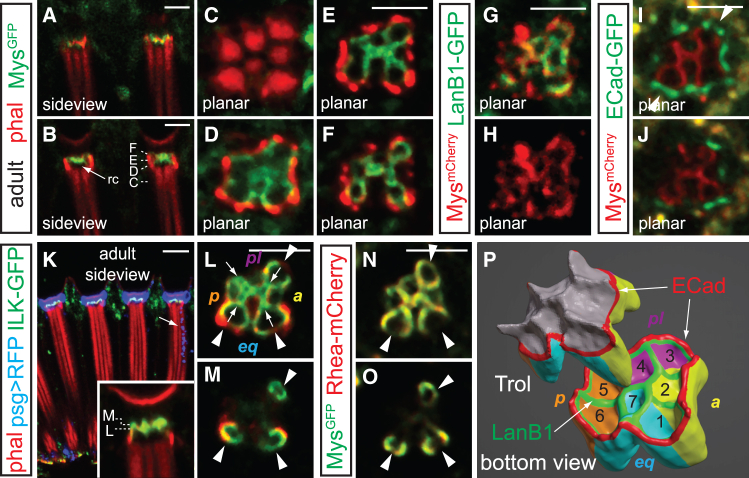
Cone cells contact rhabdomere caps via integrin Confocal micrographs of adult *mys*^*GFP*^ (A–F, phalloidin stained), *mys*^*mCherry*^, *LanB1-GFP* (G, H), *mys*^*mCherry*^, *ECad-GFP* (I and J), *psg>mRFP, ILK-GFP* (K–M, phalloidin stained), and *mys*^*GFP*^*, Rhea-mCherry* (N, O) retinas. (A–F) Grazing (A) and mid-ommatidial (B) side views, along with cross-sections (C–F; depths indicated in B), show integrin is localized on cone cell basal surfaces in contact with rhabdomere caps. (G–J) Mys^mCherry^ overlaps with LanB1-GFP grid (G; Mys shown alone in H) and is surrounded by ECad arches (I and J). (K) A side view shows that ILK decorates the cone cell surfaces bordering the rhabdomere caps. The cone cells are labeled by *psg>mRFP* (blue; *psg-GAL4* is also active in R7; arrow indicates one example). (L–O) Cross-sections show ILK-GFP (L, M; depth indicated in inset) and Rhea-mCherry (talin, N, O) co-localizing with integrin and encircling individual rhabdomere caps, with gaps at photoreceptors 1, 3, and 6 and a hollow centerline in the H-shaped figure that delineates the boundary of the four cone cells (a, anterior; eq, equatorial; p, posterior; pl, polar). Anterior is to the right. Scale bar, 5 μm. (P) A rendered bottom surface view shows Trol-filled caps and the corresponding Mys-decorated cone cell surfaces. The four cone cell surfaces and their interfaces with the Trol caps are color-labeled accordingly (numbers indicate photoreceptors with corresponding caps), with the locations of ECad arches (red) and the LanB1 grid (green) indicated.

Dome-like rhabdomere caps provide a substantial surface for potential adhesive ECM contact, and consistent with their LanB1-rich composition, *mys*^*GFP*^ and *mys*^*mCherry*^, which contain endogenous β-integrin (*myospheroid*) fluorescently tagged with GFP or mCherry at the C-terminus,^25^ decorate the underside of the cone cell quartet contacting the caps (Figures 3A–3J; this contact is referred to as the crown) and spatially overlap with LanB1-GFP (Figures 3G and 3H). Cross-sections of *mys*^*mCherry*^, *ECad-GFP* adult retinas reveal that while a composite perimeter of integrin-labeled cone cell surface and ECad-decorated AJs surrounds the rhabdomere cap base (arrowheads, Figure 3I), only the integrin-labeled cone cell surface encloses the rhabdomere cap peaks (Figure 3J).

Additionally, ILK-GFP (Figures 3K–3M) and Rhea-mCherry (*Drosophila* talin; Figures 3N and 3O) co-localize extensively with Mys at the crown, suggesting that the integrin, paxillin, and parvin (IPP) complex and talin link the rhabdomere cap/integrin interface with the actin cytoskeleton. The central H-shaped figures in Mys^GFP^, ILK-GFP, and Rhea-mCherry signals show hollow centerlines, representing the footprints of the cone cell boundaries (Figure 3L, arrows). The circular profiles above photoreceptors 1, 3, and 6 show gaps where adhesion is shared by adjacent cone cells (arrowheads, Figures 3L–3O), as illustrated in Figure 3P. Collectively, these observations demonstrate that the distal ends of photoreceptors are anchored by multiple contacts, including photoreceptor-cone cell AJs and rhabdomere cap adhesion. Moreover, the positional specificity of the taller peaks at cone cell junctions suggests that retinal cell geometry influences the morphogenesis of this extracellular structure.

### Cone cells autonomously contribute perlecan and LanB1 to rhabdomere caps

**Figure 4:**
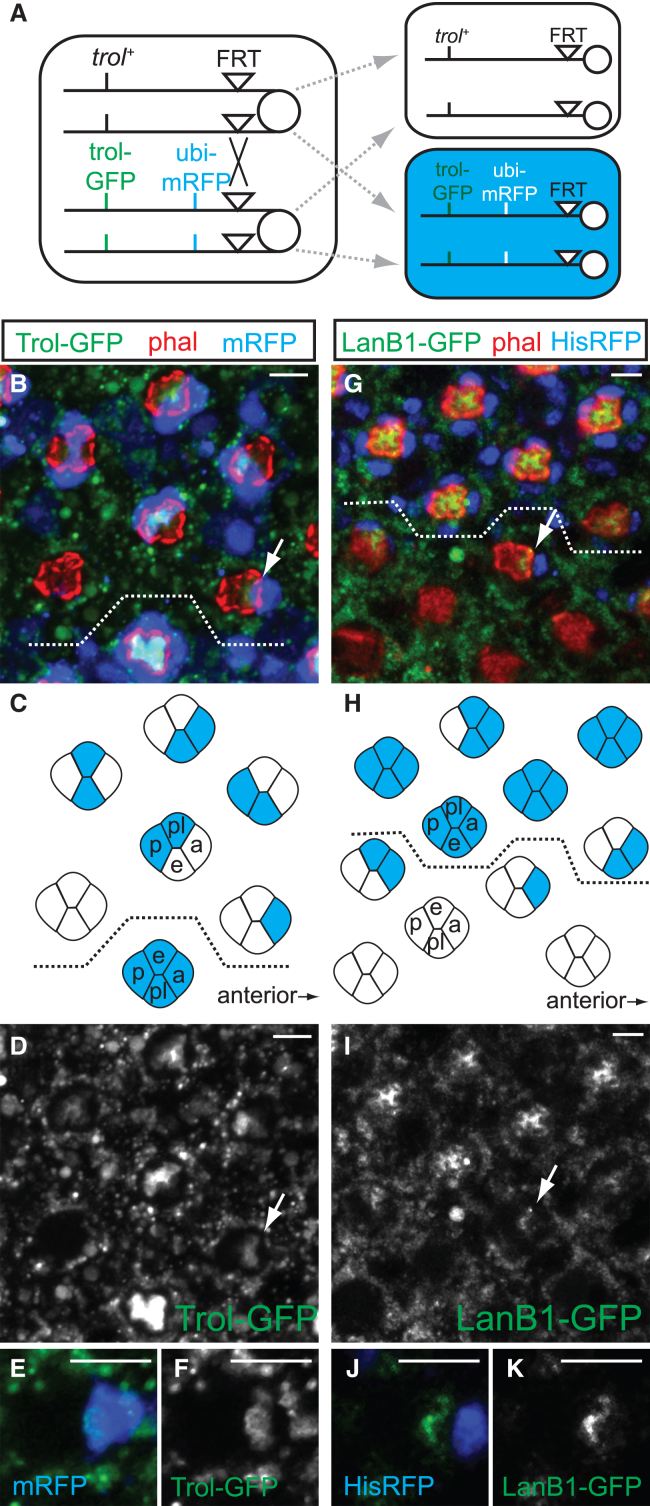
Cone cells autonomously contribute perlecan to rhabdomere caps (A) Scheme for generating mosaic retina with *FLP/FRT*-induced mitotic recombination, with *trol-GFP*^*+*^ cells labeled with mRFP (blue) expression. (B–K) Projections of *trol-GFP*, *ubi-mRFP*, *FRT*^*19*^*/FRT*^*19*^; *ey-FLP* (B–F) and *ey-FLP*; *LanB1-GFP*, *His2Av-mRFP*, *FRT*^*2A*^*/FRT*^*2A*^ (G–K) adult retinas stained with phalloidin (red). Because cone cell genotypes (mRFP) and Trol-GFP/LanB1-GFP signal are at different focal planes, B, E, G, and J are merged sub-stack projections of mRFP and GFP from different slices. Cone cell genotypes are illustrated in C and H respectively with mRFP-expressing cells shaded in blue. Trol-GFP (D and F) and LanB1-GFP (I and K) signals are separately shown, and mosaic clusters with one *mRFP*^*+*^ cone cells are indicated with arrows and shown at a higher magnification in E–F and J–K. The dash line marks the equator; anterior is to the right. Scale bar, 5 μm.

To identify the sources of rhabdomere cap components, we generated mosaic retinas in which cells were labeled genotypically for *trol-GFP* or *LanB1-GFP* with mRFP expression (*trol-GFP* scheme shown as an example in Figure 4A). If perlecan and laminin in the rhabdomere caps are deposited by neighboring cells, the presence of *trol-GFP-* or *LanB1-GFP-*negative patches (also mRFP-negative) should correspondingly diminish the GFP signal in the caps.

In *FRT*^*19A*^/*FRT*^*19A*^, *trol-GFP*, and *ubi-mRFP*^*nls*^; *ey-FLP* adult mosaic retinas, the Trol-GFP signal at the rhabdomere caps in clusters composed entirely of cone cells of one genotype appears either normal (all mRFP-positive) or completely absent (all mRFP-negative) (Figures 4B–4D). In contrast, in clusters of mixed genotypes, the contour of the Trol-GFP signal in the rhabdomere caps aligns with the mRFP-positive cone cells (also *trol-GFP*^*+*^, arrow, Figures 4E and 4F). Similarly, in *ey-FLP*; *FRT*^*2A*^*/FRT*^*2A*^, *LanB1-GFP*, *HisAV-RFP* adult mosaic retinas, the LanB1-GFP signal at the rhabdomere caps correlates with the presence of mRFP-positive cone cells (also *LanB1-GFP*^*+*^; Figures 4G–4K, arrow). These results demonstrate that both perlecan and LanB1 in the rhabdomere caps are contributed cell-autonomously by the cone cells.

### LanB1 colocalizes with Eys in late pupal retina

**Figure 5:**
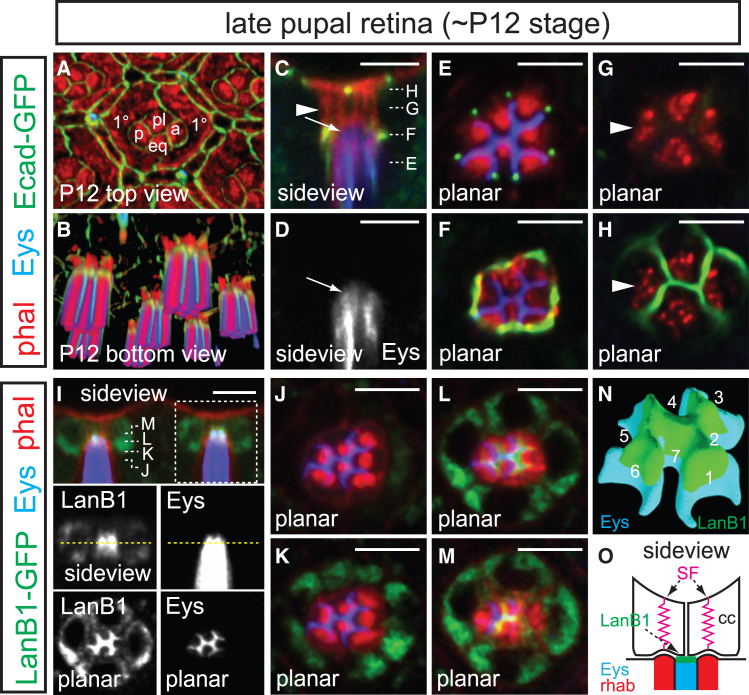
LanB1 “H” figure colocalizes with Eys in the pupal retina 3D intensity renderings (A, top view; B, bottom-sideview) and confocal sections (C–H) depict the organization of adherens junctions (*ECad-GFP*) and Eys (blue) localization in P12 pupal retina. Side views (C and D) and cross-sections (E–H; depths indicated in C) reveal Eys extends into a basal dome underneath the cone cells (C and D, arrows). Directly above this dome, longitudinal stress fibers (C, G, and H, arrowheads) connect the apical and basal surfaces of the cone cells. (I–M) Confocal micrographs show LanB1-GFP localization in relation to Eys and the “H” figure in P12 pupal retinas. Side view (I) and cross-sections (J–M; approximate depths indicated in I) show LanB1-GFP spatially overlapping with Eys within the dome. LanB1 and Eys signals in the dashed box (I) and in a cross-section at the depth indicated by the yellow dashed lines are individually shown below. Anterior is to the right. Scale bar, 5 μm. (N) A Blender model top view illustrates the colocalization of LanB1 (green) with Eys (blue) at the ECM core’s distal topography. (O) A schematic depicts the LanB1, Eys, and actin stress fiber (SF) localization at P12.

While Trol-GFP and LanB1-GFP are present at the basal surfaces of eye epithelial cells during larval and early pupal development (Figure S3),^14^ distinct structures at rhabdomere distal tips only become apparent in late pupal retinas. At P12 (dark-winged; approximately 24 h before eclosion), the localization of ECad-GFP prefigures that of adult retinas (Figure 5A), marking the AJs between photoreceptors (Figure 5E) and between photoreceptor and cone cells (Figures 5B and 5C), although there are several noticeable differences. First, the rise of pupal AJ arches is less pronounced at this stage. In addition, longitudinal stress fibers are present in an AJ arches-encircled central region of cone cells, connecting the cone cell apical and basal surfaces (arrowhead, Figures 5C, 5G, and 5H).

At P12, both the distal tips of the rhabdomeres and Eys project above the AJ arches and make contact with the basal surfaces of the cone cell quartet without any appreciable gap (arrow, Figures 5C–5F), indicating that the formation of rhabdomere cap peaks does not commence until the last ∼24 h of pupal development. As the IRS extends above the AJ arches, Eys tapers toward the apex, forming a topography reminiscent of a mountain range, with an H-shaped central ridge linking three prominent, round peaks (Figure 5N).

At this stage, LanB1-GFP is prominently localized within the cone cell cytoplasm (Figures 5I–5M; Video S4), and extracellular LanB1 and perlecan (Figure S4A–S4E; Video S5) are found at the tips of the Eys’s topography within a pocket beneath the cone cells. In cross-sections, LanB1 and Trol exhibit an H-shaped pattern, with the crossbar marking the equatorial and polar cone cell contacts and the end arms marking the junctions with the anterior and posterior cone cells (Figures 5I, 5L, and S4D). The LanB1/Trol H figure is outlined by ILK-GFP (Figures S4F–S4I; Video S6), demonstrating that this pupal precursor of the rhabdomere caps is already linked to integrin-decorated cone cell surfaces, establishing contact between rhabdomere tips and cone cells before the formation of the final cap peaks.

Video S4. LanB1 localization at P12, related to Figure 5 3D rendering (Volocity) of a P12 (dark-winged pupal) *LanB1-GFP* cluster stained with phalloidin (red) and αEys antibody (blue).

Video S5. Perlecan localization at P12, related to Figures 5 and S4 3D rendering (Volocity) of a P12 *trol-GFP* cluster stained with phalloidin (red) and αEys antibody (blue).

Video S6. ILK localization at P12, related to Figures 5 and S4 3D rendering (Volocity) of a P12 *ILK-GFP* cluster stained with phalloidin (red).

### Eys acts upstream of LanB1 and Trol in rhabdomere cap formation

**Figure 6:**
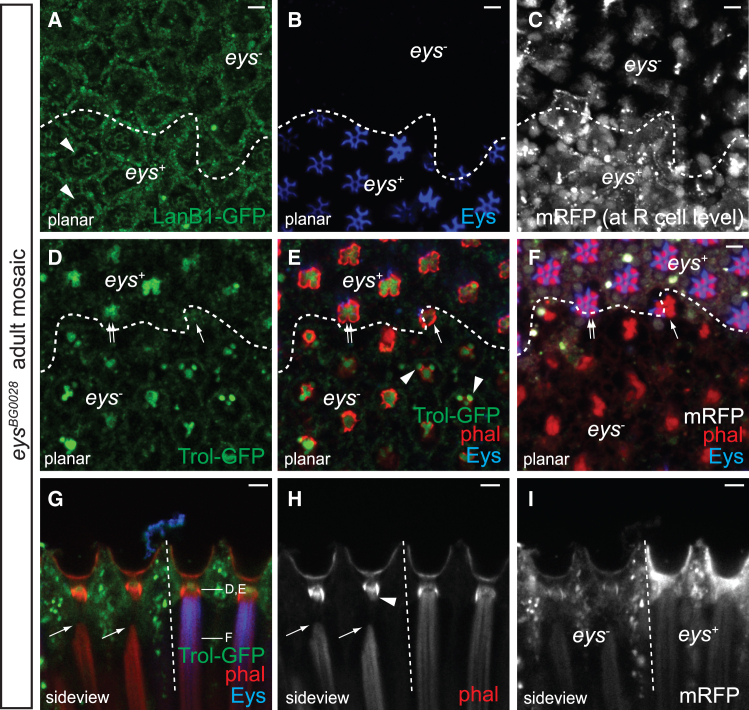
Rhabdomere Cap Formation Requires Eys (A–C) Tangential (A–F) and side-view (G–I) micrographs of adult (A–C) *ey-FLP*; *eys*^*BG02208*^, *FRT*^*40A*^*/ubi-mRFP*, *FRT*^*40A*^, *LanB1-GFP*, and (D–I) *trol-GFP*, *ey-FLP*; *eys*^*BG02208*^, *FRT*^*40A*^*/ubi-mRFP*, *FRT*^*40A*^ mosaic retinas stained with phalloidin (red) and αEys antibody (blue) are shown. In *eys*^*BG02208*^ clone, labeled by the absence of mRFP (shown in white in C, F, and I; clone boundary delineated by dashed line), LanB1-GFP (A, arrowheads) and Eys (B) are absent. (D–F) Confocal cross-sections of an *eys*^*BG02208*^ clone (approximate depths shown in G, D, and E are from the same focal plane) show aberrant Trol-GFP structures accumulating underneath the cone cells, with occasional blebbing (E, arrowheads). At the clone border, the aberrant Trol-GFP and rhabdomeric disorganization defects are less severe in mosaic clusters containing multiple *eys*^*+*^ photoreceptors (E and F). Arrow and double arrows indicate mosaic clusters with one (R6) and four (R1, 4, 5, 6) *eys*^*+*^ photoreceptors, respectively. (G–I) Side view shows rhabdomere distal tips displaced basally from the aberrant Trol-GFP structures in *eys*^*-*^ clones. The arrowhead indicates rhabdomeric fragments associated with aberrant Trol-GFP. Anterior is to the right. Scale bar, 5 μm.

The colocalization of LanB1 and perlecan with Eys at P12 suggests that Eys has a role in rhabdomere cap assembly. To test this, we examined whether Trol and LanB1 localization at the rhabdomere caps requires Eys. In adult *eys*^*BG02208*^^26^ clones, the LanB1 grid is disrupted (Figures 6A–6C), indicating LanB1 localization at the caps is Eys-dependent. Perlecan localization is also Eys-dependent; however, instead of decreasing, Trol-GFP accumulates in irregular, phalloidin-encircled structures beneath the cone cells in *eys*^*BG02208*^ clones (Figure 6D). Despite these changes, AJs remain intact (Figure S5), though side views show that the rhabdomere distal tips detach in *eys*^*BG02208*^ clones, displacing basally by 9.3 ± 1.0 μm (*n* = 8; from three independent eys mosaic retinas) (arrows, Figures 6G–6I).

Consistent with the idea that Eys is deposited by photoreceptors, the severity of these phenotypes correlates with the number of *eys*^*+*^ photoreceptors in mosaic clusters. At the clone border, Trol-GFP appears normal at the rhabdomere caps when four photoreceptors are *eys*^*+*^ (double arrow), while Trol-GFP accumulates abnormally in clusters with only one *eys*^*+*^ photoreceptor (arrow, Figures 6D–6F). Collectively, these results suggest that rhabdomere cap assembly is guided by photoreceptor-derived Eys, which acts upstream to pattern LanB1 and perlecan deposited by cone cells.

### Disruption of LanB1 grid causes rhabdomeric breakage and detachment

**Figure 7:**
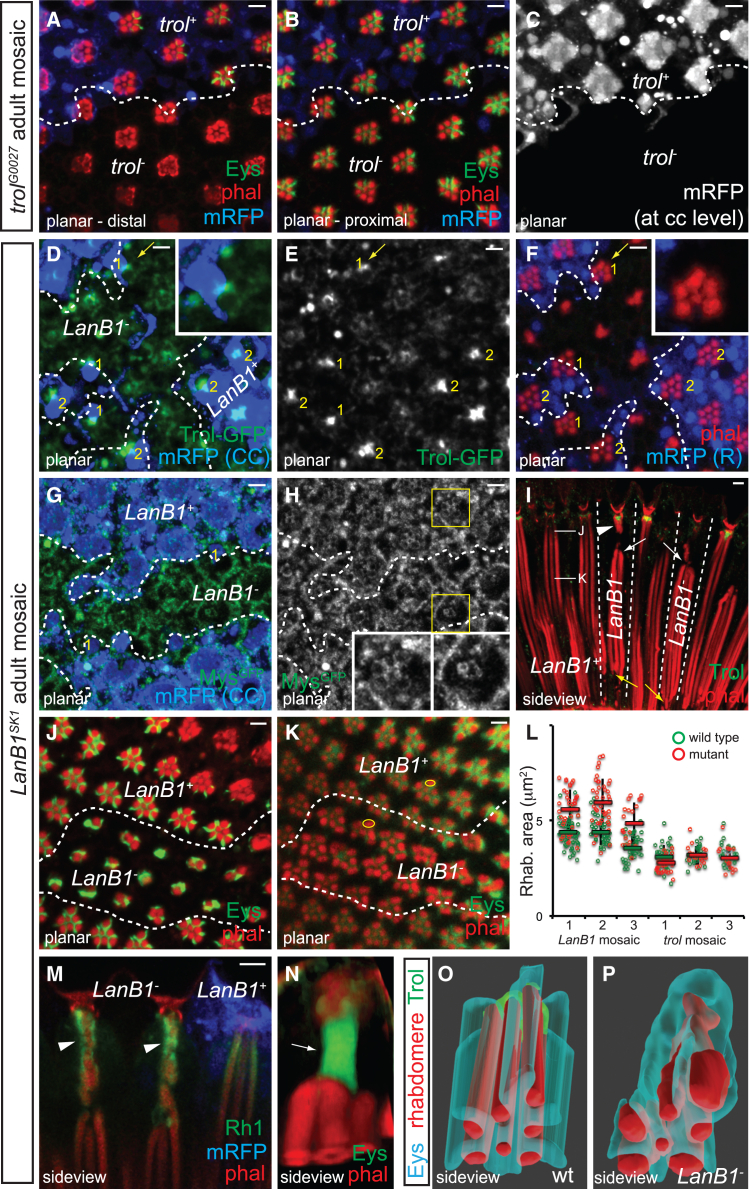
Loss of LanB1 grid causes rhabdomere detachment and fragmentation Images of adult (A–C) *trol*^*G0027*^, *FRT*^*19*^*/ubi-mRFP*, *FRT*^*19*^; *ey-FLP*, (D–F, I–K) *trol-GFP*, *ey-FLP*; *LanB1*^*SK1*^, *FRT*^*40A*^*/ubi-mRFP*, *FRT*^*40A*^, and (G–H) *mys*^*GFP*^; *LanB1*^*SK1*^, *FRT*^*40A*^*/ubi-mRFP*, *FRT*^*40A*^; *ey-FLP* mosaic retinas stained with phalloidin (red) and αEys antibody (green in A and B, J and K) are shown. (A–C) In *trol*^*G0027*^ clones, indicated by the absence of mRFP (blue; shown in white at the cone cell level in C; clone boundary delineated by dashed line), the rhabdomere and Eys patterns appear normal. (D–F) Trol-GFP and distal rhabdomeric organization are irregular in *LanB1*^*SK1*^ clones (Trol-GFP signal from D is shown separately in E, and a basal cross-section of the same mosaic retina is shown in F). Numbers in yellow (1 or 2) indicate the number of *LanB1*^*+*^ cone cells in mosaic clusters at the clone border. A cluster with one *LanB1*^*+*^ posterior cone cell is indicated by yellow arrow and shown in the inset. (G and H) The Mys^GFP^ grid pattern is disrupted in *LanB1*^*SK1*^ clones (Mys shown separately in H, with examples of wild type and *LanB1*^*SK1*^ clones highlighted in yellow boxes and shown in the insets). (I–K) Side view (I) and cross-sections (J and K; approximate depths indicated in I) show rhabdomere tips detach from both the distal (white arrows) and basal (yellow arrows) sides in *LanB1*^*SK1*^ clones. Arrowhead indicates rhabdomeric fragments associated with aberrant Trol-GFP. Anterior is to the right. Scale bar, 5 μm (L) A scatterplot shows that the average cross-sectional area of outer photoreceptor rhabdomeres (examples shown as yellow circles in K) in the proximal region in *LanB1*^*SK1*^ clones (5.6 μm^2^) is ∼35% larger (*p* value = 0.006, Student’s t test, two tailed) than in wild type (4.2 μm^2^; green and red circles representing measurements from *mRFP*^*+*^ and *mRFP*^*-*^ cells from 3 *LanB1*^*SK1*^ adult mosaic retinas). As a control, measurements from 3 *trol*^*G0027*^ adult mosaic retinas are included, and the area averages in *trol*^*G0027*^ (3.02 μm^2^) and wild type (3.2 μm^2^) clones are comparable (*p* value = 0.261, Student t test, two tailed). (M) Sideview of *LanB1*^*SK1*^ adult mosaic retina stained with αRh1 antibody (green). (N) A rendered *LanB1*^*SK1*^ cluster stained with αEys. (O and P) ImageJ 3D Viewer surface rendering of wild type (O) and *LanB1*^*SK1*^ (P) clusters viewed from below.

To understand how ECM components at the caps support rhabdomere tip tethering, we examined rhabdomere organization in *trol* and *LanB1* mutants. Adult mosaic retinas containing clones of *trol*^*G0271*^, a loss-of-function mutation in perlecan,^27^^,^^28^ showed normal Eys localization and rhabdomere organization (Figures 7A–7C), indicating that perlecan is dispensable for IRS formation and rhabdomere tethering.

In contrast to perlecan’s apparent non-essential role, *LanB1*^*SK1*^, a CRISPR-Cas9-generated allele with a frameshift deletion in the LanB1 ORF (NIG, Japan), disrupts the localization of perlecan, integrin, and Eys, as well as rhabdomere organization (phenotypes summarized in Figures 7O and 7P and Video S7). LanB1-GFP rescues the lethality associated with *LanB1*^*SK1*^, confirming that this chromosome does not contain unrelated lethal mutation.

Video S7. *LanB1* mosaic retina, related to Figure 7 3D rendering (Volocity) of a *LanB1*^*SK1*^ mosaic retina stained with phalloidin (red) and αEys antibody (green).

In adult *LanB1*^*SK1*^ clones, Trol-GFP appears irregular (Figures 7D and 7E), demonstrating that LanB1 patterns deposited perlecan. Additionally, the grid-like pattern of Mys^GFP^ is disrupted (Figures 7G and 7H, insets), supporting a notion that LanB1 interacts with and localizes integrin on the cone cell surfaces. Consistent with LanB1 being deposited by the cone cells, the severity of these defects decreases in mosaic clusters containing at least one *LanB1*^*+*^ cone cell (number of *LanB1*^*+*^ cone cells indicated in yellow, Figures 7D–7F). Moreover, since secreted LanB1 remains close to its source, the phenotypic severity varies with the position of *LanB1*^*+*^ cone cell within the cluster. For example, in mosaic clusters with a *LanB1*^*+*^ posterior cone cell (yellow arrow, Figures 7D–7F, inset; Video S8), distal rhabdomere organization in the posterior half appears normal, but the R3 rhabdomere tip—farthest away from the *LanB1*^*+*^ cell—is consistently found at a lower focal plane, highlighting the spatial dependence of LanB1 support.

Video S8. A *LanB1* mosaic cluster, related to Figure 7 3D rendering (Volocity) of a *LanB1*^*SK1*^ mosaic cluster containing a *LanB1*^*+*^ posterior cone cell (blue) stained with phalloidin (red). Because the R3 rhabdomere is farthest away from this *LanB1*^*+*^ cone cell, the R3 rhabdomere distal tip displaces basally.

Similar to *eys*^*-*^ clones, rhabdomere distal tips detach and shift basally in *LanB1*^*SK1*^ clones, displacing by 12.7 ± 2.3 μm (*n* = 9; from three independent *LanB1* mosaic retinas). Side views reveal that rhabdomere basal tips also detach, demonstrating that both rhabdomere cap- and CCP-mediated tethering requires LanB1 (arrows, Figure 7I). Consistent with the notion that these tips detach after the IRS formation, rhabdomere organization and Eys distribution are affected in a depth-specific manner. In distal cross-sections, the radial Eys pattern becomes disordered, appearing as irregular patches adjacent to misaligned rhabdomeres (Figure 7J), but approximately normal Eys organization recovers proximally (Figure 7K). The average outer rhabdomere cross-section area in the proximal region of *LanB1*^*SK1*^ clones increases by approximately 35% (Figure 7L), consistent with a reduced longitudinal tension exerted on rhabdomeres due to loss of tethering. Rhabdomere detachment is also observed in late pupal *eys*^*BG02208*^ and *LanB1*^*SK1*^ clones (Figure S6), confirming that LanB1 in the H-shaped configuration at P12 is essential in anchoring rhabdomeres.

An average of 3.8 ± 0.95 (*n* = 41; from three independent *LanB1* mosaic retinas) discrete phalloidin-positive structures (arrowheads, Figures 7I and 7M) are present underneath the aberrant Trol-GFP in adult *LanB1*^*SK1*^ clones. Phalloidin-positive structures are also seen at the same location in *eys*^*BG02208*^ clones (arrowheads, Figure 6H), though they could not be accurately counted in fluorescent images due to the lack of IRS separation. These structures are Rh1-positive (Figure 7M), suggesting that they are rhabdomeric fragments derived from breakage at distal rhabdomere ends, which may explain the presence of detached rhabdomeres. Consistent with the idea that distal fragments and detached rhabdomeres were once linked, they share a continuous IRS in *LanB1*^*SK1*^ clone (arrow, Figure 7N). Together, these phenotypes indicate that not only does LanB1 transmit longitudinal forces to align rhabdomeres, but it also protects the IRS lumen and rhabdomere distal ends from structural damage during retinal morphogenesis.

## Discussion

Our analysis of rhabdomere caps reveals that the accurate focusing of compound eye facet lenses onto the precise photodetector lattice is achieved through a unique collaboration between two cell-specific ECMs: Eys, an apical ECM proteoglycan of the interphotoreceptor matrix secreted by photoreceptors, and laminin, a basal ECM deposited by lens-forming cone cells. During retinal patterning in late larval and early pupal stages, photoreceptors and cone cells create a lumen, which is subsequently filled with these two ECMs. Remarkably, the planar contour of Eys is imprinted upon laminin, translating spatial information encoded during early pattern formation into a central three-dimensional structure as the eye develops. This process constructs a rigid, adhesive ommatidial core, which is crucial for distributing the longitudinal tension that shapes the rhabdomeres and aligns them with the optical axis.

In contrast to basement membrane assemblies, which combine ECM from multiple, often distant sources (e.g., hematocytes and hemolymph),^14^^,^^29^^,^^30^ the ECM components of this ommatidial core are derived solely from photoreceptors and cone cells. The lumen forms as cone cells close over the photoreceptors, inpocketing their apical tips (the future rhabdomeres) beneath cone cell lateral membranes.^31^ Subsequently, the basal endfeet of the cone cells migrate centrally, intercalating beneath the photoreceptor apices to form the CCP, the rhabdomeres’ proximal anchor.^9^ Thus, the ommatidial core is a closed cavity isolated from general circulation, providing a simplified, genetically accessible crucible for dissecting complex ECM/ECM and cell/ECM interactions. In addition, the tension for rhabdomere alignment acts along a single longitudinal axis across hundreds of identical units, complementing the multidirectional dynamics that drive tissue morphogenesis in other systems.^32^^,^^33^^,^^34^

Although the ECMs in the ommatidial core share a common space, they exhibit distinct mobilities. At a mid-pupal stage, Eys secreted from a specialized photoreceptor apical domain (the stalk membrane) diffuses to inflate the IRS.^10^^,^^11^ Consistent with this mobility, the Eys contour in mosaic clusters does not strictly correlate with the photoreceptor genotypes (Figures 6E and 6F). In contrast, both Trol-GFP and LanB1-GFP contours in mosaic clusters correlate with cone cell genotypes, indicating that cone cells fill the rhabdomere caps with perlecan and LanB1 that do not diffuse far from their source. This immobility resembles IOCs’ deposition of collagen at the retinal floor, where collagen remains associated with IOC cell surfaces to form composite grommets.^14^ The limited perlecan and LanB1 mobility suggests that the taller peaks at cone cell junctures are composite structures with ECMs from distinct cone cells. Nevertheless, perlecan must diffuse to fill the rhabdomere caps, and it is possible that other ECM proteins constrain its movement. Supporting this, Trol-GFP blebbing in *eys*^*-*^ clones implies increased perlecan mobility in the absence of constraints.

The rhabdomere caps contain perlecan and LanB1 but lack collagen. Although perlecan is prominent in rhabdomere caps, its exact function remains elusive; mutants show no phenotype in confocal imaging. It is likely, though not demonstrated, that perlecan contributes to the amorphous ECM of the rhabdomere caps first observed in electron microscopy.^35^^,^^36^^,^^37^ In larger flies (*Calliphora*), Seitz observed a refractive index in rhabdomere caps greater than that of the surrounding cone cell cytoplasm and comparable to rhabdomeres,^38^ suggesting total internal reflection channels light focused on the caps to the rhabdomeres below. Homogenously distributed perlecan may contribute to this elevated refractive index.

The absence of collagen distinguishes rhabdomere caps from the fenestrated membrane at the retinal floor, where collagen provides structural strength to transmit planar tension for basement area reduction.^14^ As ECM compositions cater to specific functions, the collagen’s absence at rhabdomere caps may be integral to linking rhabdomere tips to cone cell surfaces. Consistently, the CCP, which couples rhabdomere basal tips to cone cell endfeet, has an identical ECM composition.^39^ Alternatively, the collagen-less rhabdomere caps may provide an elastic yet flexible connection that secures rhabdomere tips to the rigid lens array while accommodating light-driven rhabdomere shortening^40^ and steering of the entire rhabdomere array by extraocular muscles.^41^

Genetic analysis suggests that rhabdomere cap assembly, like ECM deposition in several basement membranes,^42^^,^^43^ occurs in a stepwise manner. Perlecan localization at the caps depends on *eys* and *LanB1*, placing these two genes upstream of *trol*. However, the relationship between eys and LanB1 is more challenging to determine because the loss of either disrupts both the IRS and the LanB1 grid. Notably, while *LanB1*-deficient clones disrupt the rhabdomere caps and the IRS immediately below, IRS organization recovers deeper (25–30 microns) beneath the crown. This depth-specific disruption suggests that the LanB1 grid reinforces the distal ommatidial core against centripetal constriction caused by Poisson’s effect (a phenomenon that a viscoelastic cylinder experiences radial constriction when stretched longitudinally). Thus, it is unlikely that LanB1 regulates Eys deposition, and we place *eys* upstream of *LanB1* in rhabdomere cap assembly.

Morphological observations suggest rhabdomere cap assembly proceeds in two phases. First, cone cells secrete LanB1, which assembles in a central “H” pattern. During late pupal stages, LanB1 colocalizes with Eys, as the IRS extends into a pocket beneath the cone cells, suggesting Eys shapes the “H” pattern. Indeed, the absence of the LanB1-GFP grid in *eys*^*-*^ clones confirms that Eys is required for LanB1 localization. Eys may concentrate LanB1 by confining it to specific spaces or directly binding to LanB1, similar to how cell surface receptors recruit laminin in nascent basement membranes.^44^ Regardless of the mechanism, these findings highlight the collaboration between photoreceptor and cone cells in rhabdomere cap assembly.

In the second phase, the LanB1 grid organizes the extracellular space above rhabdomere tips into interconnected peaks, forming the rhabdomere caps. Co-localization and phenotypic results suggest that integrins anchor cone cell membranes to the LanB1 grid, forming a framework over which the rhabdomere caps rise. Stress fibers connecting the grid to cone cell apical surfaces organize at P12, suggesting that longitudinal tension is applied during this critical period of cap formation to lift cone membranes to form peaks. Meanwhile, LanB1/integrin-mediated adhesion counteracts this tension, forming the rhabdomere cap base. This model implies these caps both propagate tension for retinal morphogenesis and are shaped by that same tension.

In addition to Mys, ILK, and talin delineate the LanB1 grid, suggesting that integrins and their associated proteins connect rhabdomere caps to cytoskeletal elements within cone cells. Our previous work^14^ demonstrated that rhabdomeres in *ilk*^*-*^ clones selectively detach from the retinal floor, implying the existence of ILK-independent mechanisms that provide supplementary adhesion at the distal side. Supporting this, a fence of *ECad-GFP* arches surrounds the rhabdomere caps, likely contributing ILK-independent adhesion at the distal photoreceptor-cone cell interface. Despite the presence of other adhesion mechanisms, rhabdomere caps are indispensable for ensuring alignment by securing the distal tips. Although AJ junctions remain intact in *eys*^*-*^ clones, rhabdomere distal tips still detach, indicating that these AJ arches cannot substitute for the structural role of the caps.

Mutational analysis reveals that LanB1 is essential for rhabdomere stretching along the optical axis. In the absence of LanB1, rhabdomeres splinter at their distal ends and detach due to disrupted tethering. Furthermore, the collapse of the distal IRS lumen in *LanB1*^*-*^ clones suggests that IRS integrity is crucial for transmitting tension during alignment. At P12, LanB1 localized to Eys’s distal tips mediates the tethering of rhabdomere distal tips, suggesting that the LanB1 grid at the apex of the IRS lumen is the prerequisite for rhabdomere anchoring. Taken together, beyond facilitating adhesion between rhabdomere tips and integrin-decorated cone cell surfaces, the LanB1 grid plays a dual role. It caps the ommatidial core and imparts ECM rigidity to counteract the centripetal constriction associated with Poisson’s effect. By maintaining the distal IRS lumen, the LanB1 grid immobilizes rhabdomere distal tips, ensuring collective tethering and the even distribution of longitudinal tension necessary for precise alignment.

Mutations in human EYS (RP25) are a major cause of autosomal recessive retinitis pigmentosa, characterized by progressive photoreceptor loss.^45^^,^^46^ As Eys orthologs are absent in mice and rats, research on Eys function relies on studies in Zebrafish and flies. Zebrafish Eys localizes to the connecting cilium, where mutations cause progressive photoreceptor loss and actin disruption.^47^^,^^48^^,^^49^ Our findings reveal a role of Eys in forming the LanB1 grid, suggesting that human EYS may stabilize the photoreceptor architecture by collaborating with laminins in the interphotoreceptor matrix.^50^

### Limitations of the study

While integrins and their associated complexes mediate contact between the cone cell basal surface and rhabdomere caps, the mechanism by which the caps engage rhabdomere tips remains unclear. Similarly, how Eys contributes to the patterned organization of the LanB1 grid is not yet resolved. Eys contains five laminin G (LamG) domains, which often mediate glycan-dependent interactions; indeed, recent findings show that Zebrafish Eys binds matriglycan in a glycosylation-dependent manner, a binding essential for its retinal localization.^51^ Whether LamG domains in Eys directly influence LanB1 distribution remains to be tested. Lastly, although our genetic data suggest mechanical relationships between cells and the ECM, tensile forces were not directly measured. These limitations notwithstanding, the striking phenotypes revealed here motivate future studies integrating genetic tools with quantitative biophysical approaches to dissect the mechanics of tissue-level force coordination.

## Resource availability

### Lead contact

Further information and requests for resources and reagents should be directed to and will be fulfilled by the lead contact, Henry C. Chang (hcchang@purdue.edu).

### Materials availability

*Drosophila* lines generated in this study are available from the lead contact without restriction.

### Data and code availability

• All data reported in this paper will be shared by the lead contact upon request.
• This paper does not report original code.
• Any additional information required to reanalyze the data reported in this paper is available from the lead contact upon request.

## Acknowledgments

We thank Drs. Nick Brown (Cambridge University), Tiffany Cook (Wayne State University), and Richard Carthew (Northwestern University) for providing fly lines and Dr. Andrew Zelhof (Indiana University) for αEys antibody. This work was supported by Purdue Research Refresh Award (to H.C.C.) and 10.13039/100000002NIH (EY 10306 to D.F.R.). Funding for the LSM710 was provided by 10.13039/100000002NIH NCRR Shared Instrumentation grant 1 S10 RR023734-01A1.

## Author contributions

D.F.R. and H.C.C. designed and performed the experiments, analyzed the results, and wrote the manuscript.

## Declaration of interests

The authors declare no competing interests.

## STAR★Methods

### Key resources table

**Table:** 

REAGENT or RESOURCE	SOURCE	IDENTIFIER
**Antibodies**		

Mouse monoclonal anti-Eys	Developmental Studies Hybridoma Bank	Cat# 21A6; RRID: AB_528449
Mouse monoclonal anti-rhodopsin 1 (Rh1 *Drosophila*)	Developmental Studies Hybridoma Bank	Cat# 4C5; RRID: AB_528451
Mouse monoclonal anti-Fasciclin III (*Drosophila*)	Developmental Studies Hybridoma Bank	Cat# 7G10 anti-Fasciclin III; RRID: AB_528238
Rabbit polyclonal anti-vkg	Van De Bor et al.^29^	N/A
Goat anti-mouse secondary antibody, Alexa Fluor 488	Invitrogen	Cat# A11001; ARID AB_143167
Goat anti-rabbit secondary antibody, Alexa Fluor 568	Invitrogen	Cat# A11011; ARID AB_143157

**Experimental models: Organisms/strains**		

*D. melanogaster*: cn[1] bw[1]	Bloomington Drosophila Stock Center	RRID:BDSC_264
*D. melanogaster*: vkg::GFP: vkg[G00454]	Morin et al.^23^	FlyBase: FBti0153267
*D. melanogaster*: Fas3-GFP: Fas3^MI03674-GFSTF.1^	Bloomington Drosophila Stock Center	RRID:BDSC_59809
*D. melanogaster*: α-Cat-GFP: α-Cat^MI02577-GFSTF.0^	Bloomington Drosophila Stock Center	RRID:BDSC_59405
*D. melanogaster*: ilk-GFP: ilk[ZCL3111]	Bloomington Drosophila Stock Center	RRID:BDSC_6831
*D. melanogaster*: trol[ZCL1700]	Kyoto Drosophila Stock Center	KDSC: 110807; FlyBase: FBti0129820
*D. melanogaster*: LanB1-GFP (fTRG00681.sfGFP-TVPTBF)	Vienna Drosophila Resource Center	VDRC: v318180; FlyBase: FBst0491674
*D. melanogaster*: eys[BG02208]	Bloomington Drosophila Stock Center	RRID:BDSC_12661
*D. melanogaster*: trol[G0271]	Kyoto Drosophila Stock Center	KDSC: 111803; FlyBase: FBst0314579
*D. melanogaster*: LanB1[SK1]	National Institute of Genetics Fly Stocks	NIG-Fly:M2L-2371; Flybase: FBst1078655
*D. melanogaster*: LanB1[SK7]	National Institute of Genetics Fly Stocks	NIG-Fly:M2L-2372; Flybase: FBst1078656
*D. melanogaster*: LanB1[KG03456]	Bloomington Drosophila Stock Center	RRID:BDSC_13957
*D. melanogaster*: FRT19A	Bloomington Drosophila Stock Center	RRID:BDSC_1709
*D. melanogaster*: FRT40A	Bloomington Drosophila Stock Center	RRID:BDSC_8212
*D. melanogaster*: FRT2A	Bloomington Drosophila Stock Center	RRID:BDSC_1997
*D. melanogaster*: ubi-mRFP[nls], FRT19A	Bloomington Drosophila Stock Center	RRID:BDSC_31416
*D. melanogaster*: ubi-mRFP[nls], FRT40A	Bloomington Drosophila Stock Center	RRID:BDSC_34500
*D. melanogaster*: His2Av-mRFP1, FRT2A	Bloomington Drosophila Stock Center	RRID:BDSC_34498
*D. melanogaster*: ey-FLP (ey-FLP on X)	Bloomington Drosophila Stock Center	RRID:BDSC_5580
*D. melanogaster*: ey-FLP (ey-FLP on third chromosome)	Bloomington Drosophila Stock Center	RRID:BDSC_5577
*D. melanogaster:* srpHemo-3XmCherry/CyO	Bloomington Drosophila Stock Center	RRID:BDSC_78358
*D. melanogaster*: Ecad-GFP	Huang et al.^19^	N/A
*D. melanogaster*: psg-GAL4	Charlton-Perkins et al.^22^	N/A
*D. melanogaster*: mys^GFP^	Klapholz et al.^25^	N/A
*D. melanogaster*: mys^mCherry^	Klapholz et al.^25^	N/A
*D. melanogaster*: rhea-mCherry	Klapholz et al.^25^	N/A

**Software and algorithms**		

Volocity	PerkinElmer	
Zen 2010	Carl Zeiss	https://www.zeiss.com/microscopy/int/products/microscope-software/zen.html
Metamorph	Molecular Devices	https://www.moleculardevices.com/products/cellular-imaging-systems/acquisition-and-analysis-software/metamorph-microscopy
ImageJ	National Institute of Health, USA	https://imagej.nih.gov/ij/
Photoshop	Adobe	http://www.adobe.com/uk/products/photoshop.html
Illustrator	Adobe	http://www.adobe.com/uk/products/illustrator.html
Excel	Microsoft	https://www.microsoft.com/en-gb/
Blender	Blender Foundation	https://www.blender.org/

**Other**		

Phalloidin, Alexa Fluor 647	Invitrogen	Cat# A22287
Phalloidin, Alexa Fluor 488	Invitrogen	Cat# A12379
Zeiss LSM 710 laser scanning confocal microscope	Carl Zeiss	https://www.zeiss.com/microscopy/en/products/light-microscopes/confocal-microscopes.html

### Experimental model and study participant details

#### *Drosophila* genetics

All fly crosses were carried out at 25°C in standard laboratory conditions. *ILK-GFP* (*ilk*^*ZCL3111*^), *Fas3-GFP (Fas3*^*MI03674-GFSTF.1*^*)*, *α-Cat-GFP* (*α-Cat*^*MI02577-GFSTF.0*^), *eys*^*BG02208*^, and all *FRT/FLP* stocks were obtained from Bloomington *Drosophila* Stock Center (Indiana, USA). *trol-GFP*^*ZCL1700*^ (*trol-GFP*) and *trol*^*G0271*^ were obtained from Kyoto *Drosophila* Stock Center, and *LanB1-GFP* (fTRG00681.sfGFP-TVPTBF) was obtained from Vienna *Drosophila* Stock Center. *Vkg::GFP* (*GFP*^*vkg-G00454*^)^52^ was a gift from Dr. Wu-Min Deng (Tulane University School of Medicine, USA), *ECad-GFP*^19^ was a gift from Dr. Richard Carthew (Northwestern University, USA), *psg-GAL4*^22^ was a gift from Dr. Tiffany Cook (Wayne State University, USA), and *mys*^*GFP*^, *mys*^*mCherry*^, and *rhea-mCherry* (Klapholz et al., 2015) were gifts from Dr. Nick Brown (Cambridge University, UK). Detailed fly genotypes and sources are listed in KRT.

To analyze LanB1 function, we obtained three *LanB1* alleles, *LanB1*^*KG03456*^, *LanB1*^*SK1*^, and *LanB1*^*SK7*^, from public stock centers. While all fail to complement each other by lethality, homozygotes of *LanB1*^*KG03456*^ (Bloomington *Drosophila* Stock Center), which harbors a P transposon inserted in the 5’ UTR of *LanB1* locus,^26^ could not be rescued with *LanB1-GFP*, suggesting that the *LanB1*^*KG03456*^ chromosome contains unrelated lethal mutation(s). In contrast, lethality associated with *LanB1*^*SK1*^ and *LanB1*^*SK7*^ (Fly Stocks of National Institute of Genetics, Japan), two CRISPR/Cas9-generated alleles containing 17 bps and 14 bps frame-shift deletions in LanB1 ORF respectively (both contain premature stops shortly after the deletions), were rescued. Consistent with this, *LanB1*^*KG03456*^ mosaic retinas exhibit significant roughness, differing from the normal exteriors shown by *LanB1*^*SK1*^ and *LanB1*^*SK7*^ mosaic eyes (not shown). Thus, we used *LanB1*^*SK1*^ and *LanB1*^*SK7*^ for our analysis and present only *LanB1*^*SK1*^ phenotypes in this manuscript for the sake of clarity.

### Method details

#### Immunostaining and image acquisition

For immunostaining, dissected retinas were fixed with 4% paraformaldehyde and permeabilized with PBS + 0.3% Triton. For primary antibody, mouse anti-Eys, anti-Rh1 (4C5), and anti-Fas3 monoclonal antibodies (Developmental Studies Hybridoma Bank, Iowa) were used at 1:50 dilution. Alexa-conjugated phalloidin and secondary antibodies were used at 1:100 and 1:100 dilutions, respectively. All images were acquired with Zeiss LSM 710 laser scanning confocal microscope.

#### Image processing and rendering

Confocal stacks were rendered using ImageJ 3D Viewer > Surface display, with a resampling factor of 4. Linear thresholding was adjusted by eye to best represent cell surfaces, balancing compromises due to the high dynamic range of some probes. For example, to visualize actin-rich stress fibers and rhabdomeres without excessive flare, thresholding may exclude some lower-intensity membrane actin captured in single confocal planes, resulting in apparent gaps in the surface. Other probes, such as LanB1-GFP with a more uniform distribution, display the actual continuity of the surface. Similarly, rendering the intense LanB1-GFP signal at the central H-figure can make the continuous but weaker LanB1-GFP signal along the perimeter arches appear fragmented, though it is indeed continuous. Among ommatidia within a stack and across samples, cell shapes are highly consistent, though minor variations are present.

To generate 3D models, 3D Viewer surface renderings were exported as WaveFront (.obj) files and imported into Blender (2.9). The Remesh modifier, which simplifies surface geometry while preserving an evenly distributed quad-based mesh, was applied before coloring with Texture Paint. While no single confocal stack contains all elements of the crown/cap assembly, "Frankenstein" models can be created by manually aligning shapes from separate stacks. Volocity (PerkinElmer) were used to render confocal stacks for 3D videos.

### Quantification and statistical analysis

#### Measurement of rhabdomere cross-section

Rhabdomeres (revealed by phalloidin staining) of outer photoreceptors in basal planes of adult *LanB1* and *trol* mosaic retinas (3 independent eye for each genotype) were manually selected, and their cross-section areas were quantified by ImageJ.

#### Statistical reporting

All statistical analysis was performed using Excel. The p values are indicated in figure legends.
