# Supplementary material for: Drosophila photoreceptor tethering by a laminin-Eys scaffold
Source: iScience. 2025 May 22;28(6):112732. doi: 10.1016/j.isci.2025.112732 (PMC12167501; doi:10.1016/j.isci.2025.112732)
Supplement: Document S1. Figures S1–S6 [file mmc1.pdf]

iScience, Volume *28*

Supplemental information

Drosophila photoreceptor tethering by a laminin-Eys scaffold

Donald F. Ready and Henry C. Chang

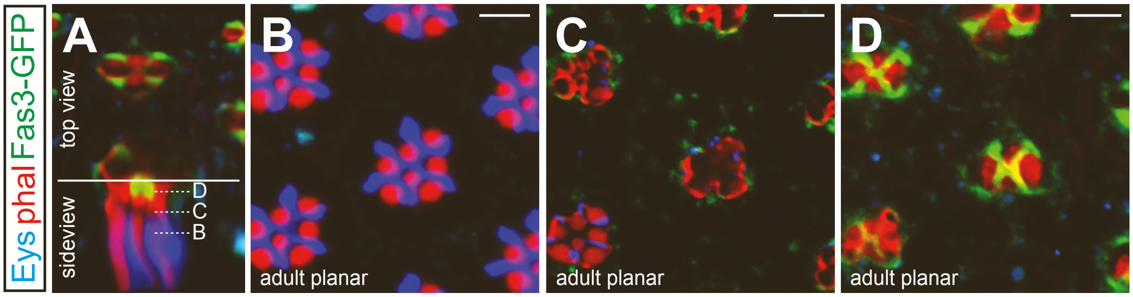

Figure S1. Septate Junction Seams the Lateral Surfaces of Cone Cells, related to Figure 1.

(A) Rendered surface view and (B-D) confocal cross-sections of a *Fas3-GFP* adult retina stained with αEys antibody (blue) and phalloidin (red) are shown. The approximate depths of these planar sections are indicated in A. Anterior is to the right. Scale bar = 5 μm.

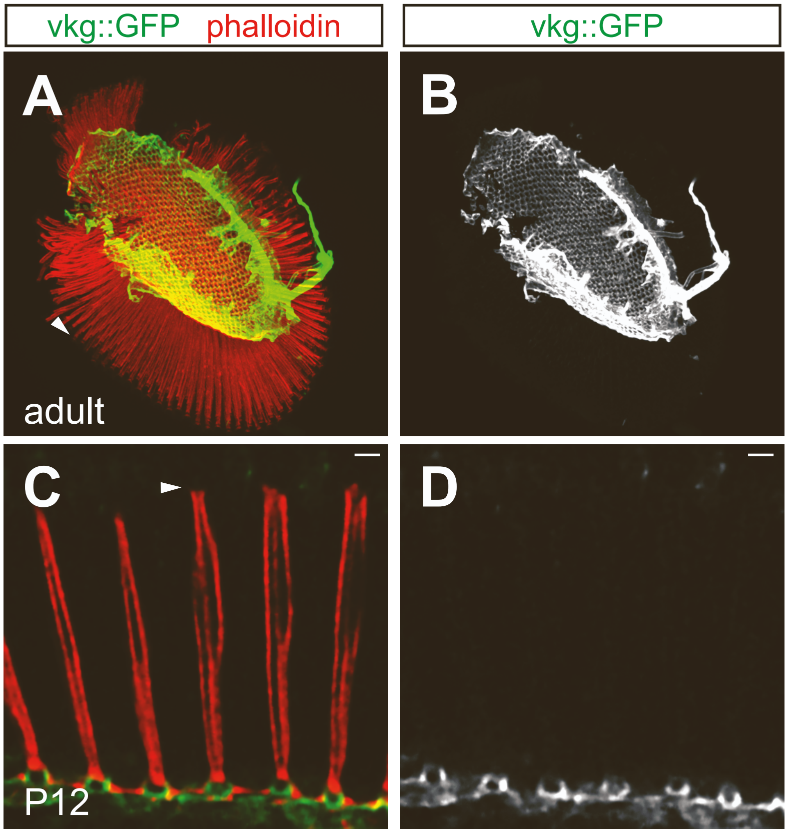

Figure S2. Collagen Is Absent at the Distal Region of Adult and Pupal Retinas, related to Figure 2.

(A-B) Projection of a *vkg::GFP* adult retina and (C-D) confocal sideview of a *vkg::GFP* pupal retina at P12 stage stained with phalloidin (red) are shown. Arrowheads indicate the position of rhabdomere caps. Scale bar = 5 μm.

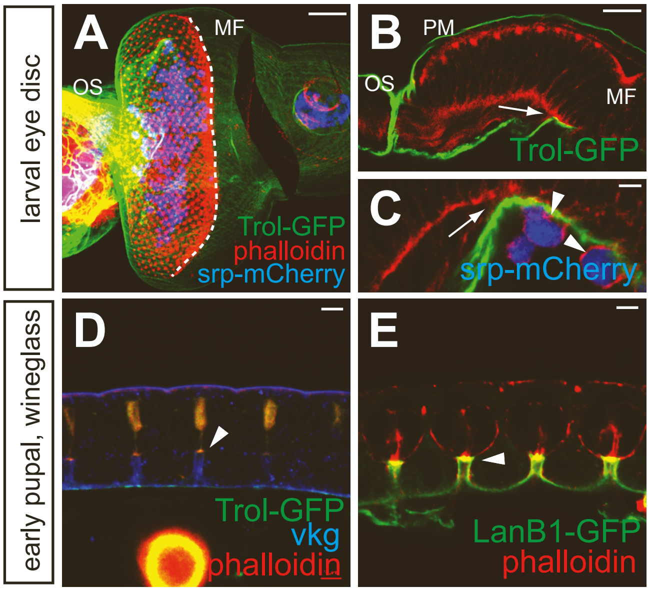

Figure S3. Perlecan and LanB1 Localizations during Early Eye Development, related to Figure 5.

(A-C) Projection (A) and confocal side views (B, C) of *trol-GFP* larval eye disc (the hemocyte marker *srp-mCherry* is included, blue) stained with phalloidin (red) are shown. Trol-GFP labels the basal surfaces of eye disc epithelium and peripodial membrane (PM), and the optic stalk (OS), which encases the photoreceptor axons connecting eye disc to the optic lobe. The Trol-labeled floor sheet detaches (arrows, B-C) from the eye disc several rows behind the morphogenetic furrow (MF, shown as a dashed line in A). srp-positive sub-retinal hemocytes (arrowheads) are present below the disc but do not express Trol-GFP at this stage. (D, E) Single confocal side views of early pupal *trol-GFP* (D) and *LanB1-GFP* (E) retinas stained with phalloidin (red) and αvkg (E, blue) antibody. At the wineglass stage, Trol-GFP and LanB1-GFP are present at the cone cell plate (arrowheads) and in sub-retinal hemocytes. Scale bar = 50 µm for (A) and 5 µm for other panels.

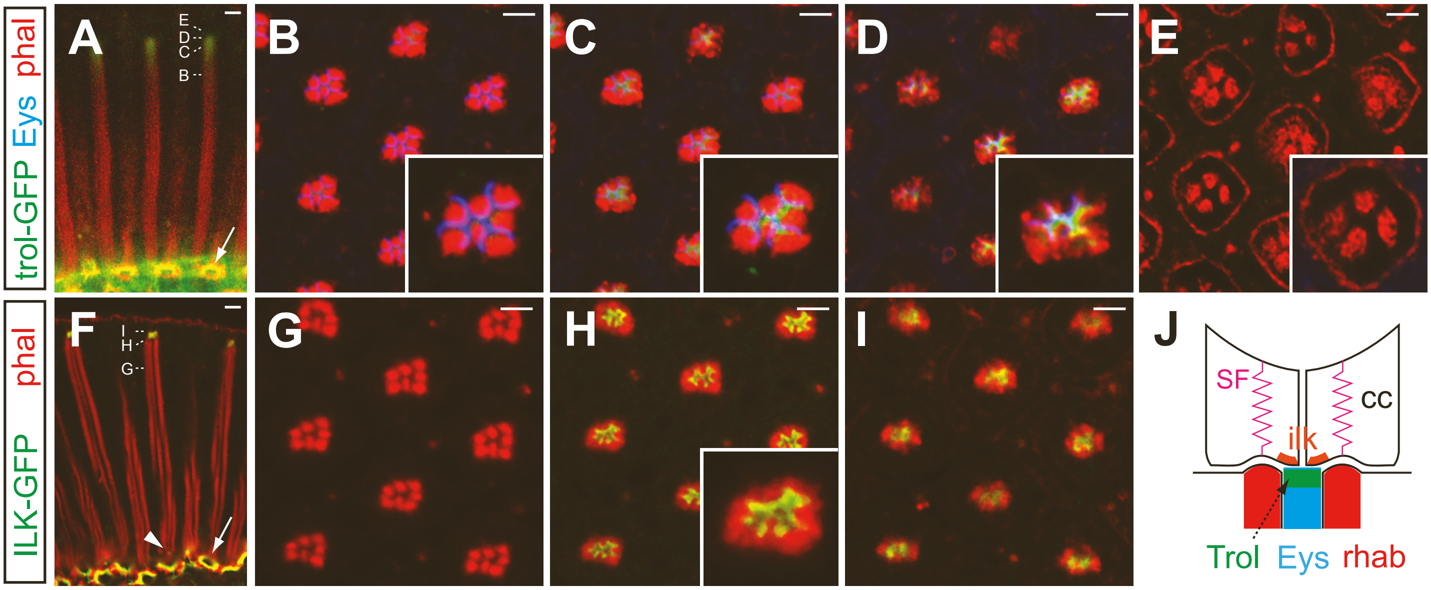

Figure S4. Perlecan Colocalizes with Eys in Pupal Retina, related to Figure 5.

Confocal micrographs of P12 *trol-GFP* (A-E) and *ILK-GFP* (F-I) retinas stained with αEys antibody (blue) and phalloidin (red). (A-E) Side view (A) and cross-sections (B-E, depths indicated in A and higher magnifications shown in insets) detail the Trol-GFP localization at the distal ends of Eys-filled IRS. (F-I) Side view (F) and cross-sections (G-I, depths indicated in F) show ILK-GFP decorating the cone cell surface adjacent to the "H" figure. Arrows indicate the association of perlecan and ILK with grommets at the retinal floor, and arrowhead indicates ILK presence at the CCP. Anterior is to the right. Scale bar = 5 μm. (J) A schematic summarizes the localizations of Trol (green) and ILK (orange) at the photoreceptor-cone cell (cc) border at P12 (rhabdomeres, red; Eys, blue; stress fibers, SF, magenta).

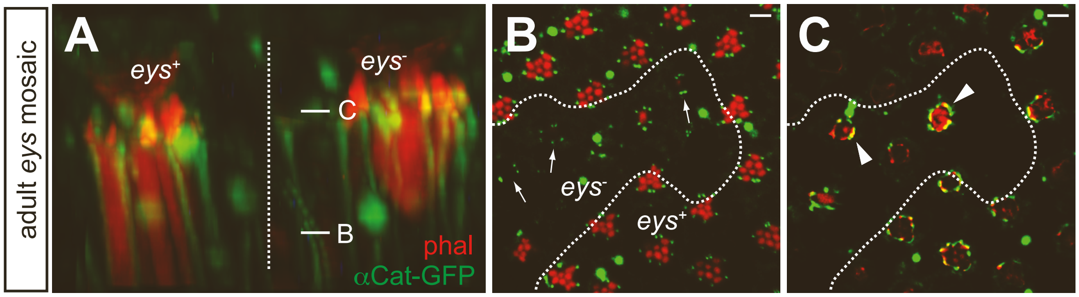

Figure S5. Loss of IRS does not Disrupt Adherens Junctions, related to Figure 6.

Rendered side view (A) and confocal cross-sections (B, C; approximate depth indicated in A) of an adult *ey-FLP; eys^{BG02208}, FRT^{40A}/ubi-mRFP, FRT^{40A}, αCat-GFP* mosaic retina stained with phalloidin (red). In *eys^{-}* clone, longitudinal AJs (αCat-GFP) between photoreceptors are still present (arrows, B) at a basal plane, where rhabdomeric material is absent due to breakage. AJs between photoreceptors and cone cells appear normal at a distal plane in *eys^{-}* clone (C, arrowheads). Dash line delineates the clone boundary. Anterior is to the right. Scale bar = 5 µm.

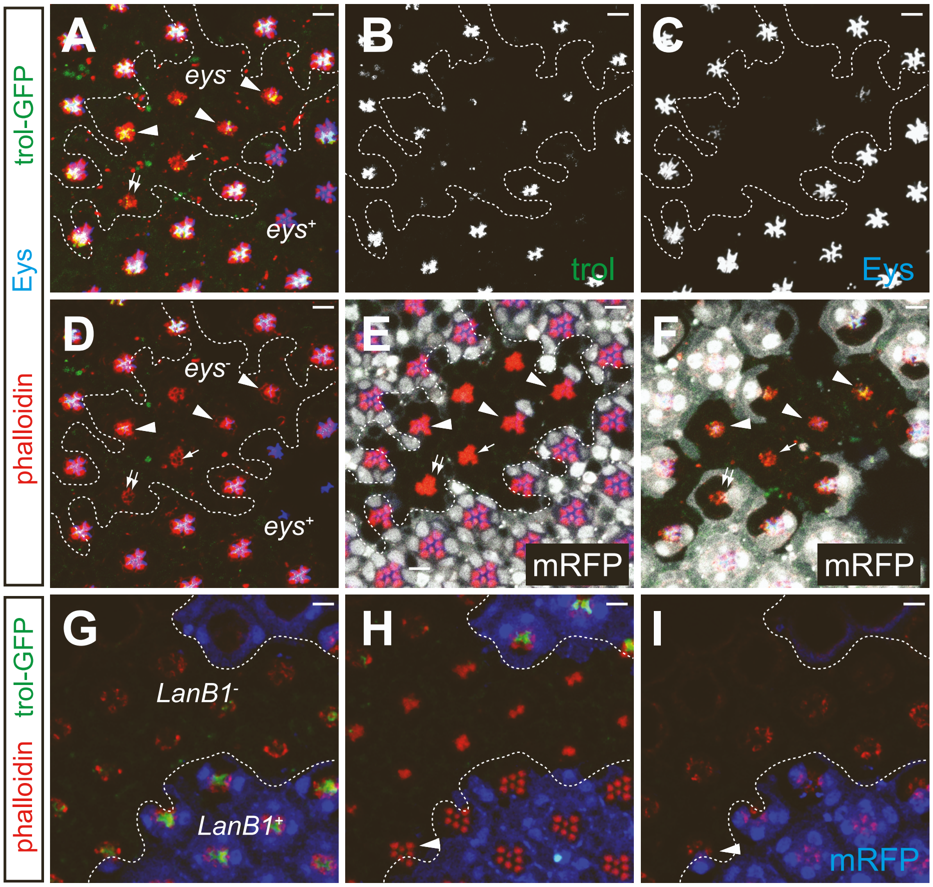

Figure S6. LanB1 "H" Figure Anchors Rhabdomere Tips at P12, related to Figures 6&7.

Micrographs of P12 (A-F) *trol-GFP, ey-FLP;* $eys^{BG02208}$*,* FRT^{40A}*/ubi-mRFP,* FRT^{40A}, and (G-I) t*rol-GFP, ey-FLP;* $LanB1^{SK1}$*,* FRT^{40A}*/ubi-mRFP,* FRT^{40A} pupal retinas stained with αEys antibody (blue) and phalloidin (red) are shown. (A-C) A maximum projection shows Trol-GFP (B) and Eys (C) are absent in the eys^- clones (dash lines are drawn based on the photoreceptor genotypes in E). (D-F) Single confocal sections of the same mosaic pupal eye showing the phenotypic severity in rhabdomere disorganization and the loss of Trol-GFP and Eys signals varies with the photoreceptor (E) but not cone cell (F) *eys* genotypes. Clusters with one eys^+ photoreceptor and no eys^+ cone cell (arrowheads) exhibit some Eys and Trol-GFP signals and mild rhabdomeric disorganization. Clusters with no eys^+ photoreceptor, regardless of the cone cell genotype (single and double arrows indicate clusters with 0 or 1 eys^+ cone cell respectively) exhibit displaced rhabdomere tips and completely lack Eys and Trol-GFP. (G-I) Cross-sections show loss of Trol-GFP (G) and rhabdomere disorganization (H) in $LanB1^-$ clones. The phenotypic severity varies with the cone cell *LanB1* genotype, as both defects are partially rescued in a cluster with one $LanB1^+$ cone cell (arrow-head). Anterior is to the right. Scale bar = 5 µm.
